# GATA6-AS1 inhibits ovarian cancer cell proliferation and migratory and invasive abilities by sponging miR-19a-5p and upregulating TET2

**DOI:** 10.3892/ol.2021.12979

**Published:** 2021-08-09

**Authors:** Hua Xu, Xiao Wang, Yinghong Zhang, Wei Zheng, Huijie Zhang

**Affiliations:** 1 Department of Obstetrics, The Affiliated Yantai Yuhuangding Hospital of Qingdao University, Yantai, Shandong 264000, P.R. China; 2 Department of Gynecology, The Affiliated Yantai Yuhuangding Hospital of Qingdao University, Yantai, Shandong 264000, P.R. China

**Keywords:** ovarian cancer, GATA6 antisense RNA 1, microRNA-19a-5p, tet methylcytosine dioxygenase 2

## Abstract

GATA6 antisense RNA 1 (GATA6-AS1) has been reported to be involved in the progression of several types of cancer. In the present study, the role of GATA6-AS1 in ovarian cancer (OC) was explored. Reverse transcription quantitative PCR was used to detect the expression of GATA6-AS1, microRNA (miR)-19a-5p and tet methylcytosine dioxygenase 2 (TET2) in OC and adjacent normal tissues. Furthermore, OC cells with GATA-AS1 either knocked down or overexpressed were established. The Cell Counting Kit-8 assay was used to evaluate cell proliferation and a Transwell assay was used to assess the migratory and invasive abilities of OC cells. A dual luciferase reporter gene assay was used to determine whether GATA6-AS1 and miR-19a-5p, and miR-19a-5p and TET2, may interact with each other. The results demonstrated that GATA6-AS1 expression level was decreased in OC tissues and cells compared with control groups. In addition, GATA6-AS1 overexpression significantly inhibited the proliferation and migratory and invasive abilities of OC cells, whereas GATA6-AS1 downregulation had the opposite effects. Furthermore, GATA6-AS1 adsorbed miR-19a-5p to repress its expression and GTA6-AS1 indirectly upregulated TET2 expression. Taken together, the findings from this study suggested that GATA6-AS1 could inhibit the proliferation and migratory and invasive abilities of OC cells via regulation of the miR-19a-5p/TET2 axis.

## Introduction

Ovarian cancer (OC) is a common tumor of the female reproductive system, and ~80% of patients are diagnosed in the first instance with advanced stage (stages III–IV) (1). In 2018, there were 22,240 newly diagnosed OC cases in the United States alone, and 14,070 patients died due to OC (2). The 5-year survival rate of OC is only 40–45% (2). It is therefore crucial to determine the underlying mechanisms of OC development and progression in order to improve diagnosis and treatment.

Long non-coding RNAs (lncRNAs) are non-coding RNAs of ≥200 nucleotides in length, which regulate a wide range of physiological and pathological processes, including gene transcription and translation, chromatin modification, cell cycle progression, cell differentiation, carcinogenesis and cancer progression (3). lncRNAs are crucially involved in the progression of various types of cancer (4–6). For example, small nucleolar RNA host gene 1 is highly expressed in non-small cell lung cancer (NSCLC) tissues and cells (4), and lncRNA activated by transforming growth factor-beta inhibits the proliferation and induces the cell cycle arrest of gastric cancer cells (5). The lncRNA GATA6 antisense RNA1 (GATA6-AS1), whose gene is located on chromosome 18q11.2, functions as a tumor suppressor in several types of cancer, such as gastric cancer (7). However, to the best of our knowledge, the role and regulatory mechanism of GATA6-AS1 in OC remain unknown.

MicroRNAs (miRNAs/miRs) are endogenous non-coding RNA molecules of ~22 nucleotides in length, which can repress the expression of target genes at the post-transcriptional level and largely participate in multiple biological processes (8). miR-19a has been implicated in the promotion of certain malignant biological behaviors, including the proliferation, metastasis and drug resistance, of cancer cells in thyroid cancer, NSCLC, colorectal cancer, osteosarcoma and OC (9–16). However, the role of miR-19a-5p in OC has not been extensively studied.

Tet methylcytosine dioxygenase 2 (TET2) is a member of the DNA demethylase TET protein family, which promotes DNA demethylation via conversion of 5-methylcytosine (5mC) into 5-hydroxymethylcytosine (5hmC). A decrease in 5-hmC levels can be used as an epigenetic marker of OC progression (17–19). However, the role of TET2 in OC remains unclear.

This study aimed to clarify the expression of GATA6-AS1 in OC, its clinical significance and mechanism. The findings may indicate that GATA6-AS1 has anticancer effects and could provide potential novel therapeutic targets for the treatment of OC.

## Materials and methods

### 

#### Tissue samples

A total of 40 patients with OC were recruited between June 2017 and March 2019 at The Affiliated Yantai Yuhuangding Hospital of Qingdao University (Yantai, China). Patients were aged between 20 and 69 years (mean age, 50.32±7.28 years). A total of 26 cases had a tumor <3 cm in diameter and 32 cases were serous and 8 cases were mucinous. According to the International Federation of Gynecology and Obstetrics staging system, 5 cases were stage I, 7 cases were stage II and 28 cases were stage III or IV. A total of 29 cases had lymph node metastases, whereas 11 did not. The present study was approved by the Research Ethics Committee of Yuhuangding Hospital of Qingdao University (approval no. 20161228A) and written informed consent was obtained from all participants. The cancerous and adjacent normal ovarian tissues were obtained during surgery and were immediately stored in liquid nitrogen until RNA extraction. Adjacent normal ovarian tissues were taken from the same patient >2 cm from the edge of the primary tumor.

#### Cell culture

The human ovarian surface epithelial HOSEPiC cell line was purchased from ScienCell Research Laboratories. The OC SKOV-3 and ES-2 cell lines were purchased from the American Type Culture Collection. The OC COC1 and A2780 cell lines were purchased from the China Center for Type Culture Collection. Cells were cultured in RPMI-1640 medium (Invitrogen; Thermo Fisher Scientific, Inc.) supplemented with 10% FBS (Thermo Fisher Scientific, Inc.) and 100 U/ml penicillin, 100 µg/ml streptomycin (Hyclone; Cytiva) and placed at 37°C in a humidified incubator containing 5% CO_2_. The medium was replaced every 2–3 days. When cells reached 70–80% confluence, they were routinely sub-cultured using 0.25% trypsin (Roche Diagnostics).

#### Cell transfection

Full-length GATA6-AS1 sequence lacking a poly-A tail was synthesized based on the sequence obtained from the National Center for Biotechnology Information database and sub-cloned into pcDNA3.1 by Shanghai GenePharma Co., Ltd. Empty plasmid, GATA6-AS1 overexpression plasmid (pcDNA3.1-GATA6-AS1), negative control short hairpin-RNA (sh-NC), shRNA targeting GATA6-AS1 (sh-GATA6-AS1), miRNA control (miR-NC, 5′-UCACAACCUCCUAGAAAGAGUAGA-3′), miR-19a-5p mimics (miR-19a-5p, 5′-AGTTTTGCATAGTTGCACTACA-3′), inhibitors control (In-NC, 5′-CACUGGUACAAGGGUUGGGAGA-3′), miR-19a-5p inhibitor (miR-19a-5p in, 5′-TGTAGTGCAACTATGCAAAACT-3′), TET2 overexpression plasmid (pcDNA3.1-TET2) and shRNA targeting TET2 (sh-TET2) were purchased from Shanghai GenePharma Co., Ltd. Lipofectamine^®^ 2000 (Invitrogen; Thermo Fisher Scientific, Inc.) was used to transfect cells according to the manufacturer's protocol. After 36 h transfection, overexpression or knockdown efficiency was examined using reverse transcription quantitative (RT-q)PCR.

#### RT-qPCR

Total RNA was extracted from cell lines or tissues using TRIzol^®^ reagent (Invitrogen; Thermo Fisher Scientific, Inc.) and was reverse transcribed into cDNA using a RevertAid™ First Strand DNA Synthesis kit (Thermo Fisher Scientific, Inc.). QuantiFast SYBR-Green PCR kit (Roche Diagnostics) was used to perform qPCR on an ABI 7300 Real-time PCR system (Applied Biosystems; Thermo Fisher Scientific, Inc.). The thermocycling protocol was as follows: Initial denaturation at 95°C for 5 min, followed by 45 repeats of a three-step cycling program consisting of 10 sec at 95°C (denaturation), 10 sec at 60°C (primer annealing) and 10 sec at 72°C (elongation), and a final extension step for 10 min at 72°C. The sequences of the primers were as follows: GATA6-AS1, forward 5′-ACCACAACCACTACCTTATGGCGT-3′, reverse 5′-TGCCATCTGGACTGCTGGACAATA-3′; miR-19a-5p, forward 5′-GTTTGCTGGGAAGGCAAAG-3′, reverse 5′-TGTTTTGCTGGGAAGGCAAA-3′; U6, forward 5′-CGCTTCGGCAGCACATATAC-3′, reverse 5′-TTCACGAATTTGCGTGTCAT-3′; TET2, forward 5′-GGACTGAGCTGCTGAATTCAACT-3′, reverse 5′-CCTCAACATGGTTGGTTCTATCC-3′; and GAPDH, forward 5′-TGTCCGTCGTGGATCTGA-3′ and reverse 5′-TTGCTGTTGAAGTCGCAGGAG-3′. The relative expression levels of GATA6-AS1, miR-19a-5p and TET2 were normalized to endogenous controls GAPDH or U6 and were expressed as 2^−ΔΔCq^ (20).

#### Cell Counting Kit-8 (CCK-8) assay

The density of cell suspensions was adjusted to 1×10^4^ cells/ml, and 100 µl of cell suspension was added to each well of a 96-well plate. After 12, 24, 48, 72 and 96 h, 10 µl CCK-8 reagent (Beyotime Institute of Biotechnology) was added to each well, and the cells were incubated for 1 h at 37°C. The absorbance was read at 450 nm using a microplate reader (BioTek Instruments, Inc.).

#### Transwell assay

OC cells were harvested using 0.25% trypsin, centrifuged at 10,000 × g for 15 min at room temperature and resuspended in serum-free medium. In the invasion assay only the membranes of the Transwell chambers (Corning, Inc.) were pre-coated with Matrigel (1:10; BD Biosciences) to mimic the extracellular matrix. The cell suspension (200 µl) containing ~5×10^4^ cells was added to the upper chamber of the Transwell chamber whereas the lower chamber was filled with 400 µl medium supplemented with 10% FBS. After incubation at 37°C for 24 h, cells that had not migrated or invaded the lower chamber were removed. The migrated or invaded cells were fixed with 4% paraformaldehyde for 10 min at room temperature and stained with 0.5% crystal violet for 5 min at room temperature. Chambers were subsequently immersed in tap water and cells were visualized and counted using a light microscope (Nikon Corporation) at ×200 magnification for five random fields.

#### Bioinformatics analysis

The GEPIA database (http://gepia.cancer-pku.cn/) was used to assess the expression of GATA6-AS1. LncBase Predicted version 2 (http://carolina.imis.athena-innovation.gr/index.php?r=lncbasev2) and TargetScan online websites (http://www.targetscan.org/vert_71/) predicted potential binding sites between GATA6-AS1 and miR-19a-5p, and between miR-19a-5p and the 3′UTR of TET2.

#### Dual-luciferase reporter gene assay

Bioinformatics analysis predicted potential binding sites between GATA6-AS1 (5′-UUUAUGUUGGUUUAAUUUCGAAAAUAAACU-3′) and miR-19a-5p, and between miR-19a-5p and the 3′UTR of TET2 (5′-ACUGGAGUCUCAUUUGCAAAACC-3′). The predicted fragment was amplified and inserted into pmirGLO Vectors (Promega Corporation) to construct the wild-type (WT) reporter vector pmirGLO-GATA6-AS1-WT or pmirGLO-TET2-WT. The mutant (MUT) reporter vectors were constructed using a GeneArt™ Site-Directed Mutagenesis system (Thermo Fisher Scientific, Inc.). The reporter vectors and miR-19a-5p mimics or miR-NC were co-transfected into OC cells, and cells were subsequently cultured for 48 h. Luciferase activity of the cells in each group was then measured using a dual-luciferase reporter assay system (Promega Corporation). *Renilla* luciferase activity was normalized to firefly luciferase activity.

#### RNA immunoprecipitation (RIP) assay

RIP assay was performed using a Magna RIP RNA-Binding Protein Immunoprecipitation kit (EMD Millipore) according to the manufacturer's protocol. In brief, cells were centrifuged at 1,500 × g and 4°C for 10 min and incubated with RIP lysis buffer, and the obtained cellular lysates were next probed with magnetic beads conjugated with a human anti-AGO2 antibody (cat. no. ab5072; rabbit polyclonal antibody; Abcam) or control IgG (cat. no. 03-110; EMD Millipore) at 4°C for 6 h. The cell lysates were then treated with proteinase K buffer (150 µl) at 55°C for 30 min to digest the protein. The magnetic beads were repeatedly washed with RIP washing buffer to remove non-specific adsorption as much as possible. The expression of GATA6-AS1 and miR-19a-5p in RIP-derived immunoprecipitated RNA was measured by RT-qPCR.

#### Western blotting

Cells were lysed with RIPA buffer (Thermo Fisher Scientific, Inc.) on ice, and the supernatant was collected after high-speed centrifugation (at 12,000 × g for 15 min at 4°C). The protein concentration was quantified using a BCA assay kit (Beyotime Institute of Biotechnology). After mixing with loading buffer, the samples were heated in a water bath at 100°C for 10 min to denature the proteins. Proteins (30 µg per lane) were separated by SDS-PAGE on 10% gels and transferred onto PVDF membranes (EMD Millipore). Membranes were blocked with 5% skimmed milk in TBST with 5% BSA at room temperature. Membranes were then incubated with primary antibodies against TET2 (cat. no. ab243323; 1:500; Abcam) and GAPDH (cat. no. ab181602; 1:2,000; Abcam) at 4°C for 8 h. Membranes were washed with TBST and incubated with secondary antibody (cat. no. ab150077; 1:1,000; Abcam) at room temperature for 1 h. Enhanced chemiluminescence reagent (EMD Millipore) was used to detect the signal on the membrane. Quantity One software v.4.62 (Bio-Rad Laboratories, Inc.) was used for densitometry analysis.

#### Statistical analysis

GraphPad Prism version 8 (GraphPad Software, Inc.) and SPSS version 16.0 (SPSS Inc.) were used to analyze the data. Data were presented as the means ± standard deviation. Whether the data were normally distributed or not was examined using the Kolmogorov-Smirnov test. For normally distributed data, an unpaired or paired t-test was used to compare data between two 2 groups. Comparisons among ≥3 groups were conducted with one-way ANOVA followed by Tukey's post hoc test. For data that were not normally distributed, comparisons between two groups were performed using paired sample Wilcoxon signed-rank test. Comparison between OC and adjacent normal tissue samples from patients with OC was performed using a paired t-test, while comparison between experimental and control groups was performed using an unpaired t-test. Correlation analyses among the expression levels of GATA6-AS1, miR-19a-5p and TET1 were performed using Pearson's correlation coefficient. P<0.05 was considered to indicate a statistically significant difference.

## Results

### 

#### GATA6-AS1 is downregulated in OC tissues

**Figure 1.**
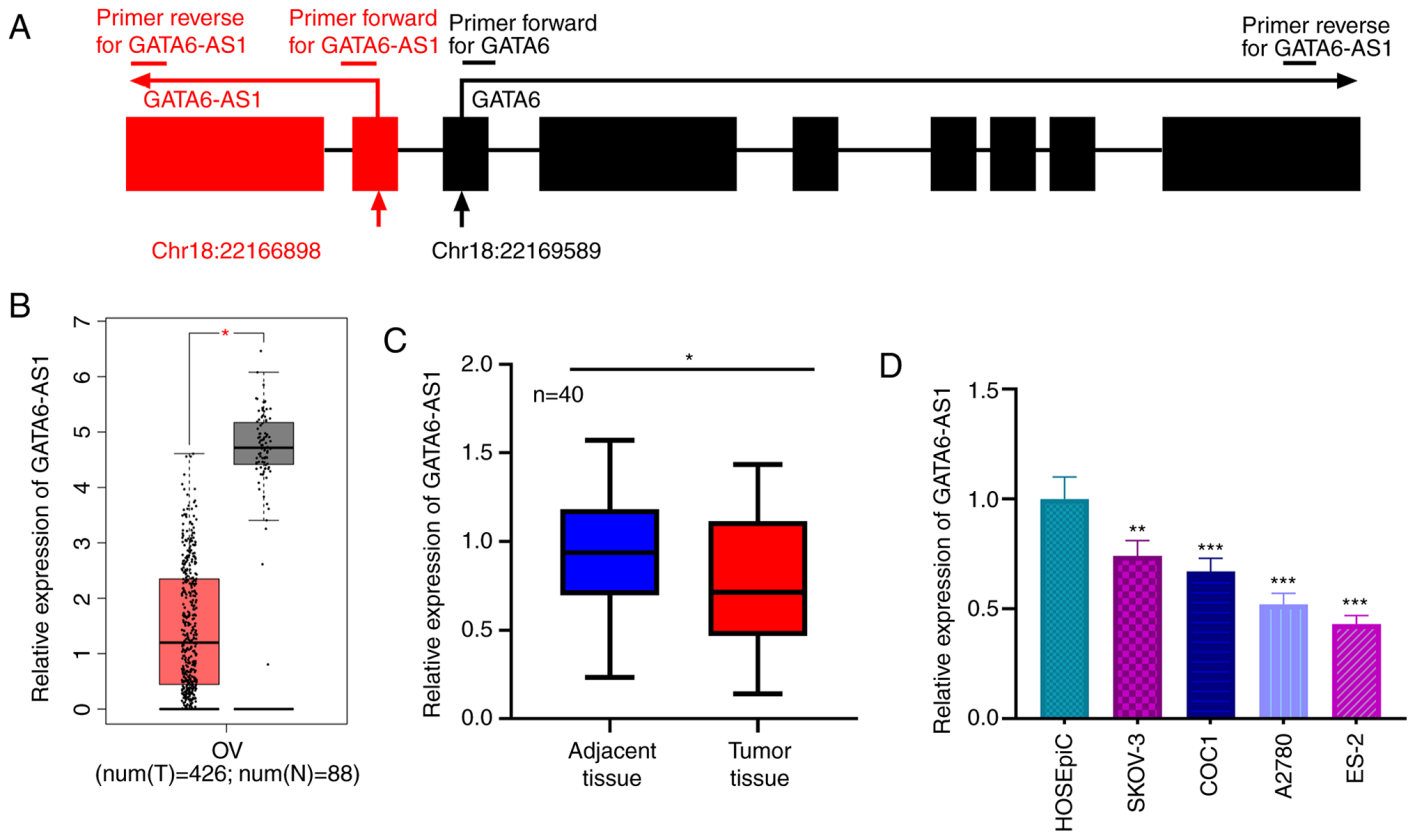
GATA6-AS1 expression is downregulated in OC. (A) Schematic diagram of the genomic location of GATA6-AS1 and GATA6 as well as exons. Red and black arrows indicate the direction of transcription of GATA6-AS1 and GATA6, respectively. (B) GEPIA database was used to analyze the expression of GATA6-AS1 in OC and normal tissues. Tumor tissues, n=426; normal tissues, n=88. (C) Expression of GATA6-AS1 in OC and adjacent normal tissues was detected by RT-qPCR. n=40. (D) Expression of GATA6-AS1 in a normal ovarian epithelial cell line and OC cell lines was detected using RT-qPCR. All experiments were performed in triplicate. *P<0.05, **P<0.01 and ***P<0.001. GATA6-AS1, GATA6 antisense RNA 1; OC, ovarian cancer; RT-qPCR, reverse transcription quantitative PCR.

GATA6-AS1 has a 1,788-nucleotide long sequence (accession no. NR_102763.1), whose gene is located on chromosome 18, next to the sequence for GATA6 (Fig. 1A). The GEPIA database was used to assess the expression of GATA6-AS1, and its expression was found to be downregulated in OC tissues relative to normal ovarian tissues (Fig. 1B). Consistent with this result, the expression of GATA6-AS1 in 40 pairs of OC and adjacent ovarian tissues was detected by RT-qPCR. The results demonstrated that GATA6-AS1 was significantly downregulated in OC tissues compared with adjacent normal tissues (Fig. 1C). Additionally, compared with HOSEpiC cells, GATA6-AS1 expression was also significantly lower in OC cell lines (SKOV-3, COC1, A2780 and ES-2; Fig. 1D). These data suggested that GATA6-AS1 may function as a tumor suppressor in OC.

#### GATA6-AS1 inhibits the proliferation and migratory and invasive abilities of OC cells

**Figure 2.**
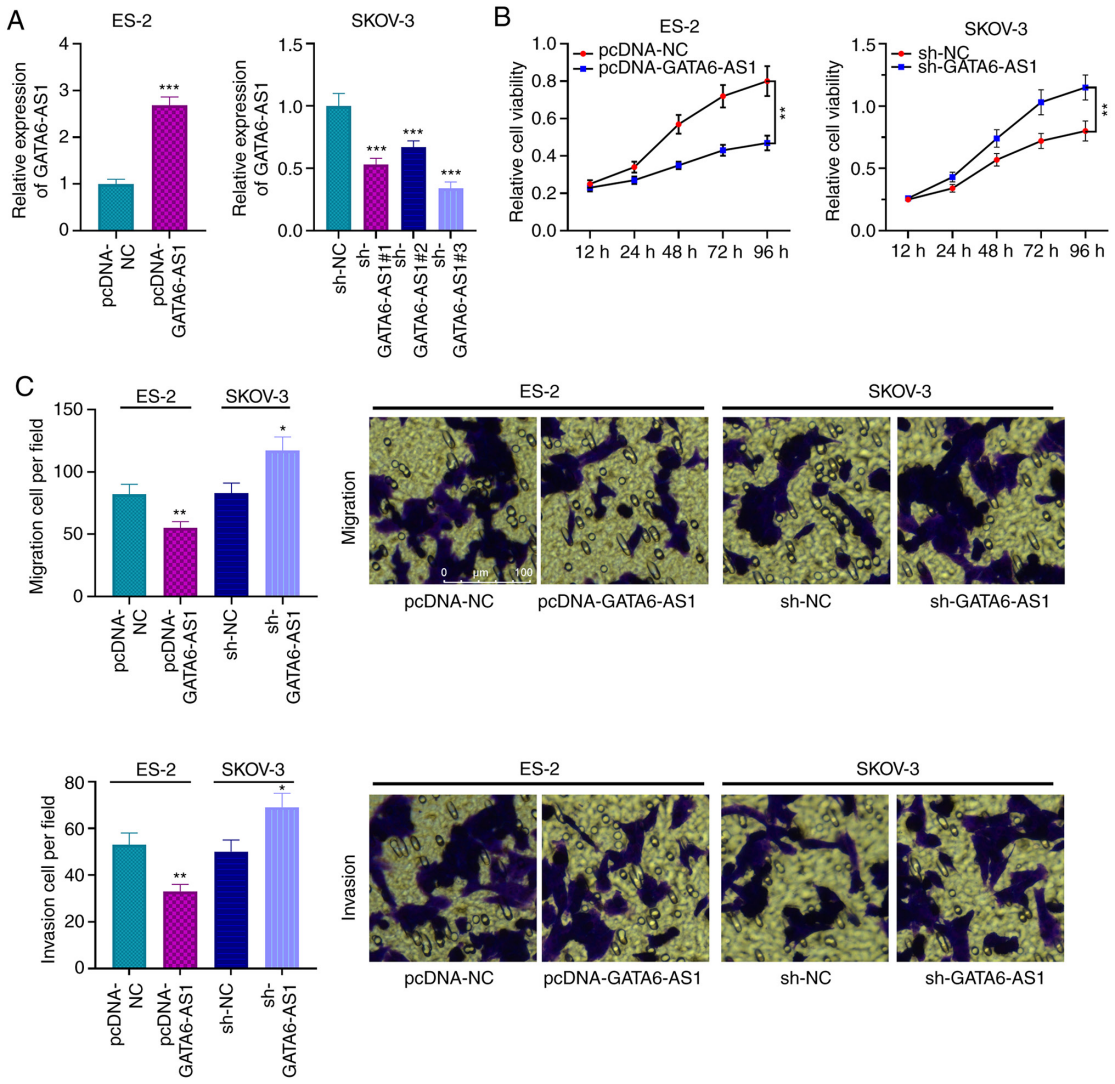
GATA6-AS1 regulates the proliferation and migratory and invasive abilities of OC cells. (A) sh-NC or sh-GATA6-AS1 was transfected into SKOV-3 cells, and pcDNA-NC or pcDNA-GATA6-AS1 was transfected into ES-2 cells. GATA6-AS1 expression was detected using reverse transcription-quantitative PCR. (B) OC cell proliferation was detected using a Cell Counting Kit-8assay. (C) OC cell migratory and invasive abilities were determined using Transwell assays. All experiments were performed in triplicate. *P<0.05, **P<0.01 and ***P<0.001. GATA6-AS1, GATA6 antisense RNA 1; OC, ovarian cancer; sh, short hairpin; NC, negative control.

The results indicated that GATA6-AS1 expression in OC cell lines (SKOV-3, COC1, A2780 and ES-2) was significantly lower compared with HOSEpiC cells (Fig. 1D). Among these four types of cell, GATA6-AS1 expression was the lowest in ES2 cells, while it was the highest in SKOV-3 cells. Subsequently, the overexpression GATA6-AS1 plasmid was transfected into ES-2 cells whereas GATA6-AS1 expression was knocked down in SKOV-3 cells using shRNA. The results from RT-qPCR confirmed that these transfections were successful (Fig. 2A). Furthermore, the results from CCK-8 and Transwell assays showed that GATA6-AS1 overexpression inhibited the proliferation and migratory and invasive abilities of ES-2 cells compared with the control group, whereas GATA6-AS1 knockdown had the opposite effects in SKOV-3 cells (Fig. 2B and C). These findings indicated that GATA6-AS1 may inhibit the malignant biological behaviors of OC cells.

#### GATA6-AS1 sponges miR-19a-5p

**Figure 3.**
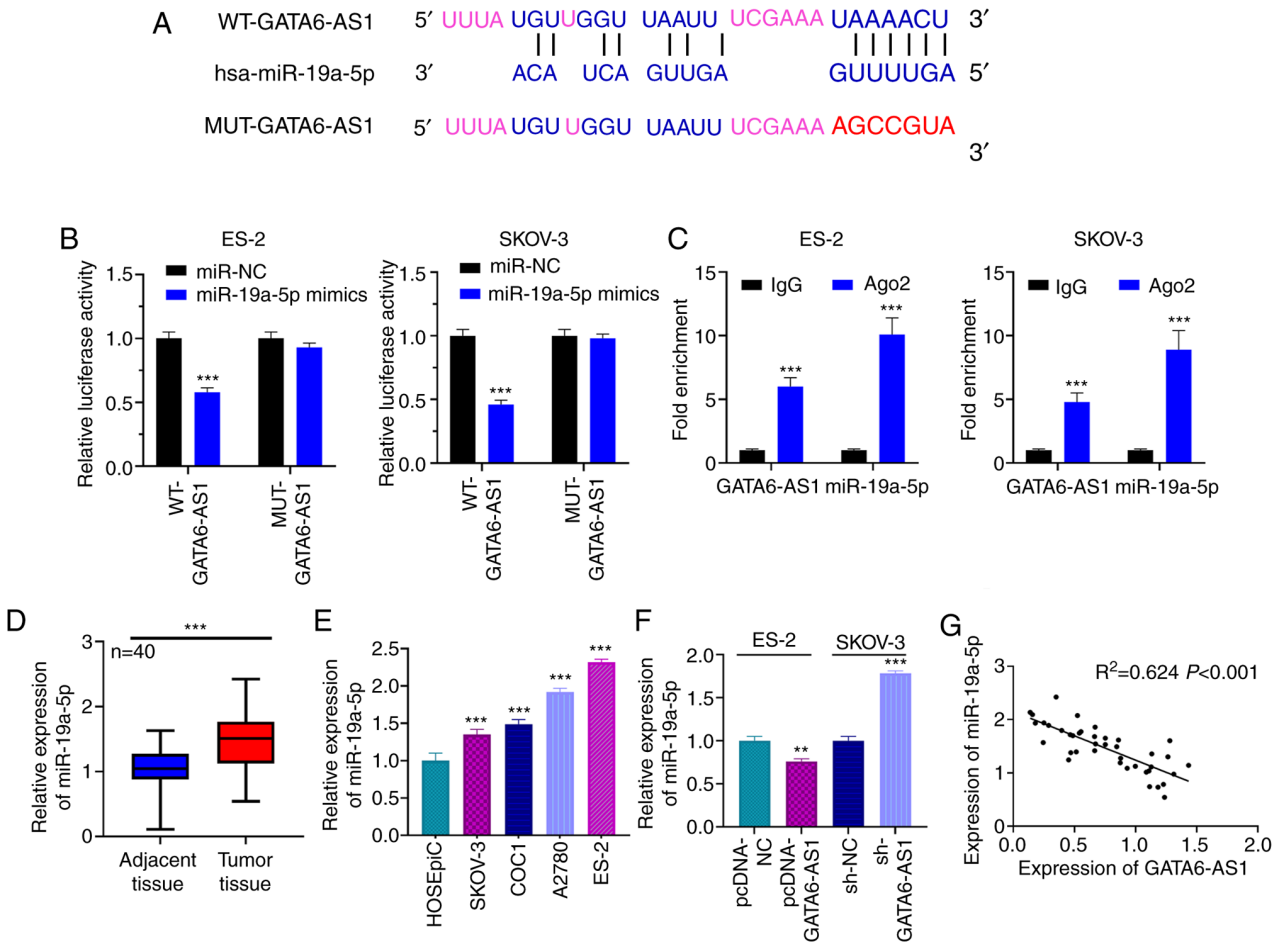
GATA6-AS1 targets miR-19a-5p and represses its expression. (A) Bioinformatics analysis was used to predict binding between GATA6-AS1 and miR-19a-5p. (B) Dual luciferase reporter gene assays were used to assess the binding relationship between miR-19a-5p and GATA6-AS1. (C) GATA6-AS1 and miR-19a-5p were co-immunoprecipitated with Ago2 in the RNA immunoprecipitation assay. (D) RT-qPCR was used to detect the expression of miR-19a-5p in OC and adjacent normal tissues. n=40. (E) Expression of miR-19a-5p in OC cell lines was detected using RT-qPCR. (F) GATA6-AS1 was overexpressed or knocked down, and the expression of miR-19a-5p was subsequently detected by RT-qPCR. (G) Pearson's correlation analysis was used to examine the relationship between GATA6-AS1 and miR-19a-5p in OC samples. All experiments were performed in triplicate. **P<0.01 and ***P<0.001. GATA6-AS1, GATA6 antisense RNA 1; OC, ovarian cancer; RT-qPCR, reverse transcription quantitative PCR; miR, microRNA; NC, negative control; sh, short hairpin; MUT, mutant; WT, wild-type.

Bioinformatics analysis was performed using LncBase Predicted version 2, and it was predicted that GATA6-AS1 contained a potential binding site for miR-19a-5p (Fig. 3A). The results from dual luciferase reporter gene assay showed that the luciferase activity of the WT-GATA6-AS1 reporter was decreased by miR-19a-5p mimics, whereas that of MUT-GATA6-AS1 reporter was not significantly affected (Fig. 3B). Furthermore, results from RIP assay suggested that GATA6-AS1 and miR-19a-5p may directly interact with each other in the immunoprecipitate containing Ago2 (Fig. 3C). In addition, miR-19a-5p expression was assessed in OC and normal adjacent tissues using RT-qPCR. The results demonstrated that miR-19a-5p expression was significantly higher in OC tissues compared with normal adjacent tissues (Fig. 3D). Furthermore, miR-19a-5p expression was significantly increased in the four OC cell lines compared with HOSEpiC cells (Fig. 3E). It was also demonstrated that GATA6-AS1 upregulation in ES-2 cells could significantly inhibit miR-19a-5p expression, whereas GATA6-AS1 knockdown in SKOV-3 cells resulted in miR-19a-5p upregulation (Fig. 3F). Pearson's correlation analysis showed that miR-19a-5p expression was negatively correlated with GATA6-AS1 expression in OC tissue samples (Fig. 3G), suggesting that miR-19a-5p may be a downstream target of GATA6-AS1.

#### TET2 is a target gene of miR-19a-5p in OC

**Figure 4.**
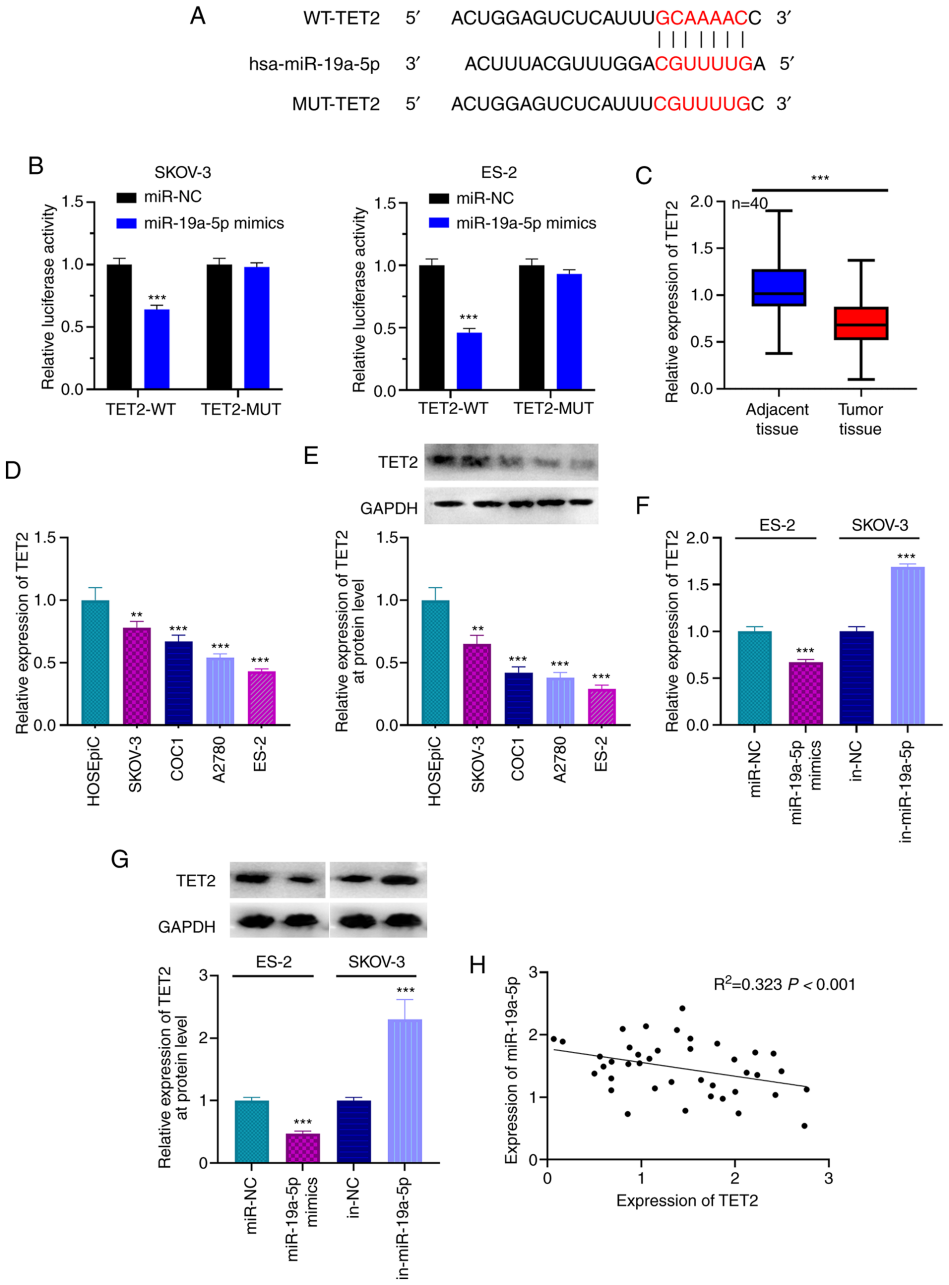
TET2 is the target gene of miR-19a-5p in OC cells. (A) TargetScan analysis predicted the presence of a binding site between the 3′UTR of TET2 and miR-19a-5p. (B) Dual luciferase reporter gene assay was used to confirm the binding relationship between miR-19a-5p and TET2. (C) Expression of TET2 in OC and adjacent normal tissues was detected using RT-qPCR. (D) Expression of TET2 in cell lines was detected using RT-qPCR. (E) Expression of TET2 in cell lines was detected using western blotting. (F) miR-19a-5p mimics and inhibitors were transfected into ES-2 and SKOV-3 cells, respectively, and the expression levels of TET2 was detected using RT-qPCR. (G) miR-19a-5p mimics and inhibitors were transfected into ES-2 and SKOV-3 cells, respectively, and the expression levels of TET2 was detected using western blotting. (H) Relationship between expression of miR-19a-5p and TET2 was examined using Pearson's correlation analysis. n=40. All experiments were performed in triplicate. **P<0.01 and ***P<0.001. miR, microRNA; OC, ovarian cancer; TET2, ten eleven translocation 2; UTR, untranslated region; RT-qPCR, reverse transcription-quantitative PCR; MUT, mutant; WT, wild-type; NC, negative control; in, inhibitor.

The candidate targets of miR-19a-5p were predicted using TargetScan, and TET2 was found to be a candidate target of miR-19a-5p. The binding site is presented in Fig. 4A. The results from RT-qPCR indicated that miR-19a-5p expression was significantly increased in ES-2 and SKOV-3 following transfection with miR-19a-5p mimics (Fig. S1). Furthermore, dual luciferase reporter gene assay showed that the luciferase activity of WT-TET2 reporter was decreased by miR-19a-5p mimics; however, miR-19a-5p mimics had no effect on the luciferase activity of MUT-TET2 reporter (Fig. 4B). In addition, results from RT-qPCR and western blotting demonstrated that TET2 expression in OC tissues and cells was significantly lower compared with that in adjacent tissues and normal ovarian epithelial cell (Fig. 4C-E). Compared with miR-NC, the expression level of miR-19a-5p was significantly increased in ES-2 cells transfected with miR-19a-5p mimics (Fig. S1A). The expression of miR-19a-5p was significantly decreased in SKOV-3 following transfection with miR-19a-5p inhibitors compared with inh-NC (Fig. S2). In addition, transfection with miR-19a-5p mimics significantly decreased TET2 mRNA and protein expression in ES-2 cells, whereas miR-19a-5p inhibitors significantly increased TET2 mRNA and protein expression in SKOV-3 cells (Fig. 4F and G). Pearson's correlation analysis demonstrated that miR-19a-5p expression was negatively correlated with TET2 mRNA expression in OC tissue samples (Fig. 4H). These findings indicated that miR-19a-5p could target and negatively regulate TET2 expression in OC.

#### GATA6-AS1 alters OC cell phenotype via the miR-19a-5p/TET2 axis

**Figure 5.**
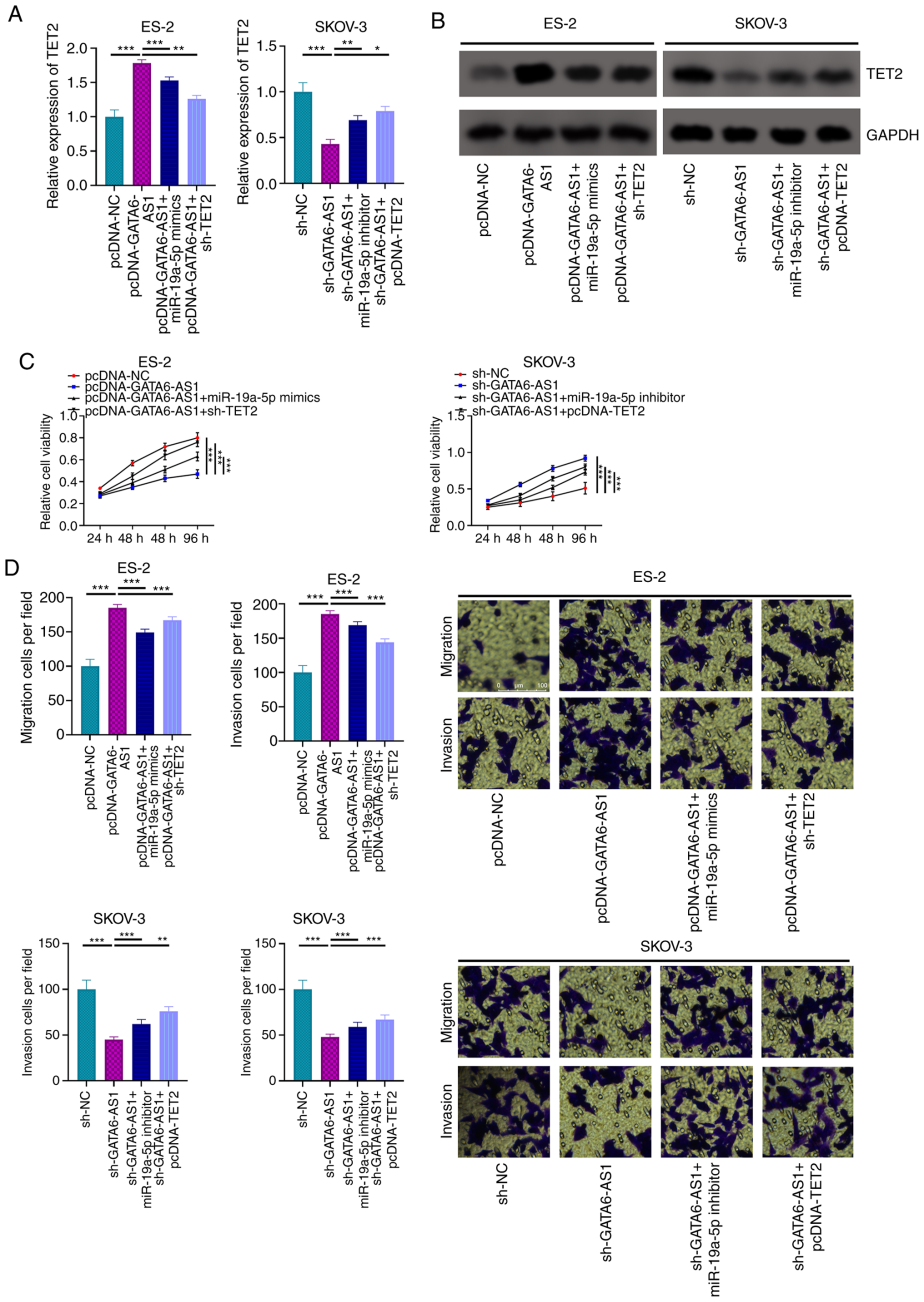
GATA6-AS1 regulates OC via a miR-19a-5p/TET2 axis. ES-2 cells were transfected with pcDNA-NC, pcDNA-GATA6-AS1, pcDNA-GATA6-AS1 + miR-19a-5p mimics or pcDNA-GATA6-AS1 + sh-TET2. SKOV-3 cells were transfected with sh-NC, sh-GATA6-AS1, sh-GATA6-AS1 + miR-19a-5p inhibitors or pcDNA-GATA6-AS1 + pcDNA-TET2. Subsequently, the mRNA and protein expression levels of TET2 in OC cells were detected using (A) RT-qPCR and (B) western blotting, respectively. (C) Proliferation of OC cells was assessed using a Cell Counting Kit-8 assay. (D) Transwell assays were used to evaluate OC cell migratory and invasive abilities. All experiments were performed in triplicate. *P<0.05, **P<0.01 and ***P<0.001. GATA6-AS1, GATA6 antisense RNA 1; OC, ovarian cancer; miR, microRNA; TET2, ten eleven translocation 2; RT-qPCR, reverse transcription-quantitative PCR; NC, negative control; sh, short hairpin.

To determine the role of the GATA6-AS1/miR-19a-5p/TET2 axis in the progression of OC, ES-2 cells were transfected with pcDNA-NC, pcDNA-GATA6-AS1, pcDNA-GATA6-AS1 + miR-19a-5p mimics or pcDNA-GATA6-AS1 + sh-TET2; and SKOV-3 cells were transfected with sh-NC, sh-GATA6-AS1, sh-GATA6-AS1 + miR-19a-5p inhibitors or pcDNA-GATA6-AS1 + pcDNA-TET2. The results from RT-qPCR and western blotting demonstrated that GATA6-AS1 overexpression resulted in increased TET2 mRNA and protein expression in ES-2 cells, and that this effect was attenuated by miR-19a-5p mimics or sh-TET2. Furthermore, GATA6-AS1 knockdown decreased TET2 expression in SKOV-3 cells, but this inhibitory effect was partially reversed by co-transfection with miR-19a-5p inhibitor or pcDNA-TET2 (Fig. 5A and B). The results from CCK-8 and Transwell assays demonstrated that GATA6-AS1 overexpression significantly inhibited the proliferation and migratory and invasive abilities of ES-2 cells; however, this inhibitory effect was partially compensated by transfection with miR-19a-5p mimics or sh-TET2 (Fig. 5B-D). Furthermore, GATA6-AS1 knockdown promoted the malignant properties of SKOV-3 cells, which was partially attenuated by transfection with miR-19a-5p inhibitor or pcDNA-TET2 (Fig. 5B-D). These results suggested that GATA6-AS1 may regulate OC progression via the miR-19a-5p/TET2 axis.

## Discussion

lncRNAs are widely expressed in various types of human tumor, and dysregulation of certain lncRNAs has been shown to be associated with tumor progression, highlighting their potential roles as biomarkers and/or therapeutic targets (21,22). GATA6-AS1 expression has been reported to be downregulated in gastric cancer, where it decreases frizzled class receptor 4 (FZD4) expression by recruiting zeste homolog 2 and inducing trimethylation at lysine 27 of histone H3 of the FZD4 promoter region, inhibiting therefore the Wnt/β-catenin signaling pathway and preventing epithelial-mesenchymal transition (7). Furthermore, downregulated expression of GATA6-AS1 activates the PI3K/AKT/Snail signaling pathway via regulation of miR-582/FOXO3 axis and subsequently promote the proliferation and metastasis of gastric cancer cells (23). The present study demonstrated that GATA6-AS1 expression was decreased in OC and that GATA6-AS1 significantly inhibited the proliferation and migratory and invasive abilities of OC cells, confirming that GATA6-AS1 may be a tumor-suppressive lncRNA in OC.

MiRNAs have been involved in the regulation of cell proliferation, differentiation and apoptosis, amongst other cell biological processes (24–29). Both miR-19a-3p and miR-19a-5p have been reported to be crucial in the promotion or inhibition of cancer progression. For example, miR-19a-3p was reported to promote the proliferation of hepatocellular carcinoma cells via regulation of the PIK3IP1/AKT pathway (30); however, another study reported that miR-19-3p induces colorectal cancer cell apoptosis by repressing the expression of Fas cell surface death receptor (31). Furthermore, miR-19a-5p expression is significantly decreased in NSCLC, and miR-19a-5p can suppress the proliferation, migration and invasion of NSCLC cells (28). In the present study, miR-19a-5p expression was significantly increased in OC tissues and cells compared with adjacent normal tissues and normal cells, respectively. In addition, functional experiments showed that miR-19a-5p reversed the tumor-suppressive effect of GATA6-AS1, suggesting that miR-19a-5p may act as an oncomiR involved in the promotion of OC progression. GATA6-AS1 was also identified as a competitive endogenous RNA of miR-19a-5p, which could sponge and reduce the expression of miR-19a-5p. This demonstration may partly explain the mechanism leading to miR-19a-5p dysregulation in OC.

TET proteins are a class of α-ketoglutarates and Fe^2+^-dependent dioxygenases that consist of three members, TET1, TET2 and TET3. All three TET proteins are capable of transforming 5mC into 5hmC, and mutation or dysregulation of TET contribute to tumorigenesis in several types of cancer (32). TET2 is the second most frequently mutated gene in hematopoiesis, and TET2 mutations were demonstrated to drive blood cell tumorigenesis (33). Defects in TET2 enzyme activity contribute to the incidence of myeloid cancer, and measurement of 5-hmC levels in myeloid cancer is a promising strategy for diagnostic and prognostic evaluation (34). As previously reported, TET2 expression is downregulated in OC and is crucial in inhibiting cancer progression (19). TET2 regulates 5mC oxidation pathways to activate or inhibit the expression of downstream genes through modulating transcription repressors or associated activators (19). However, to the best of our knowledge, there are only a few reports focusing on the underlying mechanism of TET2 dysregulation in OC. Notably, it has been reported that miR-19a-5p can negatively regulate the expression of TET2 in glioblastoma (13). In the present study, a similar regulatory mechanism between miR-19a-5p and TET2 was demonstrated in OC. In addition, GATA6-AS1 positively regulated the expression of TET2 via repression of miR-19a-5p in OC cells. The results from functional experiments suggested that the tumor-suppressive role of GATA6-AS1 in OC was partly dependent on its regulatory function on TET2. These results not only clarify the mechanism by which GATA6-AS1 could repress OC progression, but also propose a reasonable explanation for TET2 dysregulation in OC.

In summary, the present study demonstrated that GATA6-AS1 expression is downregulated in OC tissues and cells. Furthermore, the results from functional experiments showed that GATA6-AS1 could inhibit the proliferation and migratory and invasive abilities of OC cells via regulating the miR-19a-5p/TET2 axis. Restoration of GATA6-AS1 may therefore be considered as an effective strategy for OC treatment. In future studies, *in vivo* experiments should be performed to confirm the present findings. In addition, the small cohort size and lack of survival analysis were a limitation to the present study. The role of GATA6-AS1 as a prognostic biomarker should therefore be investigated in a larger cohort.

## Supplementary Material

Supporting Data

## Data Availability

The datasets used and/or analyzed during the present study are available from the corresponding author on reasonable request.
