# Supplementary material for: GATA6-AS1 inhibits ovarian cancer cell proliferation and migratory and invasive abilities by sponging miR-19a-5p and upregulating TET2
Source: Oncol Lett. 2021 Aug 9;22(4):718. doi: 10.3892/ol.2021.12979 (PMC8371982; doi:10.3892/ol.2021.12979)

Figure S1. miR-NC and miR-19a-5p mimics were transfected into (A) ES-2 and (B) SKOV-3 cells and the expression of miR-19a-5p was detected by reverse transcription quantitative PCR. ***P<0.001. miR, microRNA; NC, negative control.

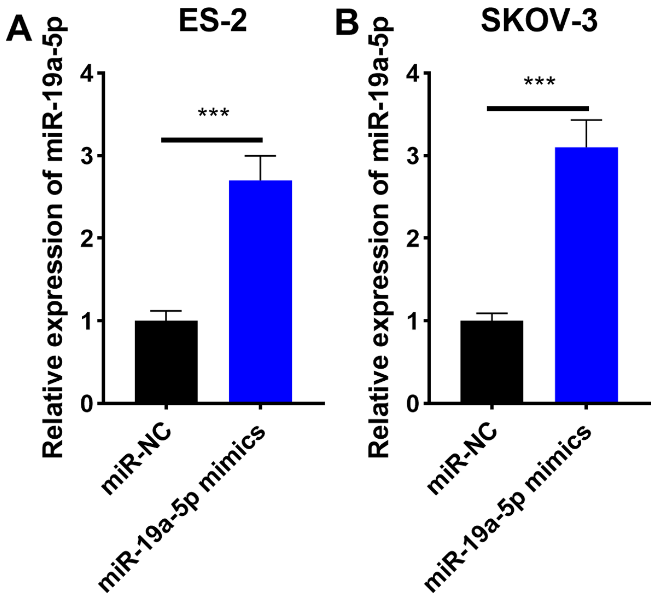

Figure S2. in-NC and miR-19a-5p inhibitors were transfected into SKOV-3 cells and the expression of miR-19a-5p was detected by reverse transcription quantitative PCR. $^{***}P<0.001$. miR, microRNA; NC, negative control; in, inhibitor.

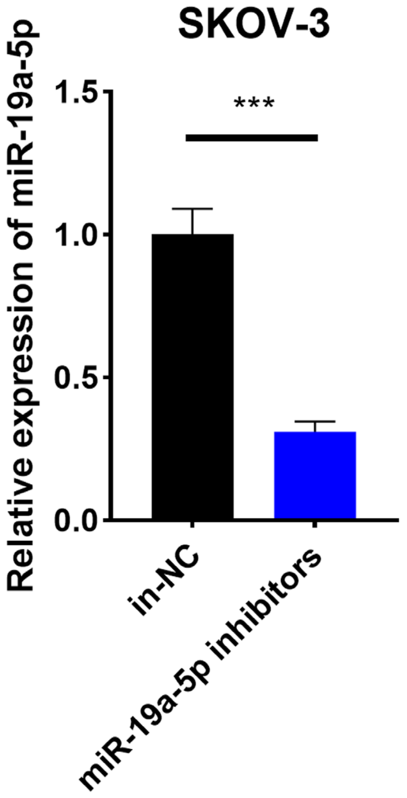

Supplement: Supporting Data [file Supplementary_Data.pdf]
